# Assessing the significance of first place and online third places in supporting Malaysian seniors’ well-being during the pandemic

**DOI:** 10.1057/s41599-023-01655-5

**Published:** 2023-04-07

**Authors:** Teck Hong Tan, Izian Idris

**Affiliations:** 1 Xiamen University Malaysia, School of Economics and Management, Bandar Sansuria, Malaysia; 2 Sunway University, Sunway Business School, Bandar Sunway, Malaysia

**Keywords:** Environmental studies, Economics, Health humanities

## Abstract

The enforced lockdowns and social distancing measures associated with COVID-19 may have influenced older adults’ preferences towards their homes and neighborhoods as well as social spaces. One objective of this research is to determine whether home and neighborhood environments (“first place”) affect how satisfied older adults are with their lives during the epidemic. This study also examined the extent to which social spaces that exist in the virtual world (“online third places”) affect older adults’ life satisfaction when they would have to practice risk-averse behaviors in times of pandemic. To collect data, this study analyzed the responses of 500 active older adults and conducted in-depth interviews with seven older adults who served as neighborhood leaders in Klang Valley, Malaysia. The study found that there is a direct relationship between older adults’ satisfaction with their current housing and their overall life satisfaction during the pandemic. Similarly, having a quality neighborhood nearby increases the likelihood of living a satisfied life during the pandemic. Most online third places, with the exception of instant messaging apps, do not appear to provide older adults with an adequate platform to interact with their friends, participate in social networking, and join communities for emotional support during the pandemic. The findings and recommendations of this study would be very useful in developing effective interventions to promote aging in place during the coronavirus outbreak.

## Introduction

Most older adults consider their home and immediate neighborhood to be their “first place,” where they often spend most of their time. “Third places” on the other hand, include social spaces where older adults can meet and socialize. Oldenburg (1999) defines the third place as a place where a person can take a short break from the home (“first place”) and the workplace (“second place”). Indoor spaces (such as restaurants, shopping malls, and places of worship) and public open spaces (such as recreational parks and other natural areas) are examples of third places. The COVID-19 pandemic has significantly changed older people’s expectations of their social spaces (“third places”). People now maintain different social contacts than in the past, in both physical (real) and online (virtual) social spaces (Langlais and Vaux, 2022). The increasing use of smartphones and technological advances, including social networking websites, mobile computing, and telecommunications networks, has had a significant impact on social spaces, as many of these social spaces have moved online. To support the needs and preferences of older people in the context of these new circumstances, policymakers and community leaders must understand and adapt to these changes.

Promoting healthy lifestyles in the built environment should be part of the overall change process to revitalize older adults’ living spaces. To improve the well-being of older adults, the home and neighborhood environments (“first place”) should keep pace with these many changes by creating a supportive residential environment for the changing needs of older adults that allows them to age in their place longer. Prior to the pandemic, there were studies in both Asian and Western countries on the relationship between neighborhood and housing characteristics and well-being in later life. For example, Zhang and Zhang (2017), Feng et al. (2018), and Yu et al. (2019) examined how neighborhood characteristics affect the well-being of seniors in China. Similarly, Park and Lee (2016) and Tsuchiya-Ito et al. (2019) demonstrated that higher levels of satisfaction with neighborhood environmental factors were responsible for higher well-being among older adults in Korea and Japan. In addition, Tan and Lee (2022) and Tan and Lee (2023) examined the effects of residential environment characteristics and neighborhood environment on life satisfaction among older adults in Malaysia. In Western countries, older adults’ well-being is also associated with better home and neighborhood environments (Parra et al., 2010; Curl and Mason, 2019).

In the absence of studies on the relationship between “first place” and older adults’ well-being during the pandemic, it will be interesting to see if the pandemic changes older adults’ preferences for their homes and immediate neighborhoods, as this could have an impact on their well-being.

Because the elderly are more susceptible to severe illness and death caused by the virus COVID-19, the pandemic has had a significant impact on their behavior. Due to the need to maintain a physical distance and limit contact with others, which can negatively affect well-being, many elderly people felt isolated and depressed (Gaggero et al., 2022). According to Xia and Li (2018) and Leigh-Hunt et al. (2017), pandemic-related loneliness and isolation affect 10–40% of older people in Europe and China, compared with 56% in Malaysia (Marzo et al., 2021), but this figure includes people of all ages, not just older people. It is not yet known how many Malaysian older adults suffer from loneliness and depression as a result of the pandemic.

To counteract social isolation, many older adults have used technology to keep in touch with family and friends while being advised to stay home and limit physical contact with others during the pandemic. This has led to an increase in the amount of time older adults spend using digital communication tools and other online platforms (Sixsmith et al., 2022). According to a study by Grini and Ueland (2023), 67% of seniors use communication technologies to reduce their loneliness. During the pandemic, 56% of seniors used communication technology in some other way to communicate with others (Haase et al., 2021).

In the Internet age, people form new social bonds in the virtual world, where cyberspace is an ideal social space to meet new people and form long-term relationships. Unlike most communities in the real world, these virtual communities are based on shared interests (Bugliarello, 1997). Some argue that virtual worlds can function as “online third places.” Online third places are virtual social spaces where people can interact, mingle, and form communities. Research suggests that online third places can help alleviate the social isolation and loneliness caused by the social distancing measures implemented in many countries to contain the pandemic COVID-19 (Zaccaria et al., 2020).

Online third places come in a variety of forms (Soukup, 2006; Robinson and Deshano, 2011; McArthur and White, 2016; Aldosemani et al., 2016). One of the commonly used online third places that allow users to interact and present themselves to a specific audience is chatrooms on social media. Due to the pandemic COVID-19, people are spending much more time in social media chatrooms (Mou, 2020). Social media chatrooms are widely used on Facebook, Instagram, Tiktok, Twitter, Reddit, and Xiaohongshu, among others. Online community forums and blogs, where the author or blog owner frequently posts updates, are another common type of third place. A blog contains areas where readers can post comments, which allows two-way interaction between readers and the blog author. Both senders and recipients can participate in a discussion about a particular topic in a discussion forum.

Real-time communication tools such as instant messaging applications are another type of online third place. Since they facilitate interpersonal communication, they are popular among many mobile applications (Anglano et al., 2016). In 2021, WhatsApp was the most popular instant messaging service in the world with more than 2 billion active monthly users (Ceci, 2022). WeChat, Line, Telegram, and Facebook Messenger are some other instant messaging services. According to Parkinson et al. (2021), online service communities have the potential to serve as an online third place by facilitating the exchange of social support among users. In addition to their practical or utilitarian needs, online service communities created by companies help customers meet needs beyond mere consumption, such as the need for support, friendship, and recognition (Parkinson et al., 2021). For example, sick older adults can benefit from online health service communities enabled by companies. Online third places also include online learning communities where people can meet to share information and support on a particular topic or area of interest.

Previous research has found that physical (real) third places can improve older people’s perceptions of life satisfaction in Malaysia (Tan and Lee, 2022, 2023). It is unclear whether third places created in the virtual world can affect older people’s well-being. In light of this, the significance of this paper is to examine the life satisfaction of the elderly based on their needs and preferences for online third places during the coronavirus pandemic.

## Well-being in the elderly

One of the most difficult issues facing older adults is how to improve their life satisfaction. According to Sirgy and Cornwell (2002), life satisfaction is primarily determined by the evaluation of individual life concerns. Life satisfaction is a common outcome used to assess the cognitive dimension of older adults’ well-being (Diener et al., 1999). Older adults face a variety of problems, including feelings of insecurity and loneliness and family members’ fears of obsolescence. As a result of social isolation, older adults’ well-being in terms of life satisfaction may suffer, posing serious health risks. The measurement of older adults’ well-being in this study focuses on how their home, neighborhood, and online social environment affect their life satisfaction during the pandemic.

## Home and neighborhood environments (“First place”)

Individuals’ housing needs and aspirations change throughout the stages of life. Older adults may need to reconsider their housing needs and goals, which may require some housing adjustments aimed at improving their internal housing conditions while keeping the principle of aging in place in mind. It would be interesting to think of older adults’ satisfaction with their current housing as an assessment of housing quality. Individuals’ evaluation of their housing influences how they respond to their living environment, which is critical to their well-being (Djebuarni and Al-Abed, 2000; Tan, 2012). Previous research has found that satisfaction with housing is influenced by housing characteristics such as unit size, number of bathrooms, type of housing, and size of the kitchen (Savasdosara et al., 1989; Amole, 2009; Tan, 2012). In addition to the housing characteristics mentioned above, illumination is another factor to consider in the internal built environment, as a well-lit environment provides a safe environment for older adults to move around more, thereby improving their quality of life. Kunduraci (2017) emphasized the importance of indoor lighting in preventing falls in older adults. In summary, housing satisfaction is considered an important factor in an individual’s overall well-being, and the extent to which housing conditions for older adults meet their needs can have an impact on their life satisfaction (Sigry and Cornwell, 2002).

Cerin et al. (2006) developed a neighborhood walkability scale (NEWs) to assess the performance of the immediate neighborhood environment, which includes several key environmental characteristics such as street connectivity, accessibility, esthetics, traffic and crime safety, and walking infrastructure. Among the components of neighborhood walkability, access to good environmental qualities such as a beautiful natural landscape (Ambrey and Fleming, 2014; Fleming et al., 2016) is a notable predictor of life satisfaction. Evidence suggests that well-designed open space near and between homes is necessary for a livable neighborhood that can contribute to residents’ quality of life, as interaction with neighbors or friends is considered valuable social capital that has been shown to influence life satisfaction (Maas et al. (2006)).

Accessibility, street connectivity, and pedestrian-friendly sidewalks are other factors that can influence life satisfaction (Yu et al., 2017). It is increasingly recognized that older adults who can walk to nearby amenities in a pedestrian-friendly neighborhood can age successfully (Sallis et al., 2016). Older adults who live in a pedestrian-friendly neighborhood are more likely to be more physically active, which can significantly reduce their risk of depression (James et al., 2017) and improve their life satisfaction (Ryff, 2014). On the other hand, higher levels of neighborhood problems, such as crime and traffic safety, are associated with lower individual life satisfaction (Balfour and Kaplan, 2002). It is often assumed that residents who live in a safe environment are more satisfied with their neighborhood (Tan, 2016).

In times of a pandemic, it would be crucial to pay attention to changes in the individual living environment needs. To prevent the further spread of disease, the Malaysian government has implemented various forms of lockdowns. The most restrictive form is the Movement Control Order (MCO), in which individuals are not allowed to move from their residences (Fan and Cheong, 2021). Therefore, during the outbreak, affected individuals, especially the elderly, would have to stay at home longer and stay away from public areas. This could affect older people’s expectations of internal and external environmental factors that make them feel more satisfied with life.

## Online social spaces (“Online third places”)

The social environment of older people is widely recognized as a key mechanism in explaining their well-being. Older adults should have access to a supportive social environment that enables them to maintain their physical activity and social relationships, which could improve their life satisfaction (Tan and Lee, 2022). Life after retirement is generally viewed by older adults as an opportunity to spend more time with family or friends. According to a growing body of research, third places play an important role in providing an accessible environment for older adults’ social interaction, which may improve life satisfaction (Tu et al., 2020).

Movement restrictions caused by the Covid-19 pandemic have altered the lifestyles of older people, particularly with regard to their needs and preferences for social places. During the pandemic outbreak, older adults were forced to stay at home and avoid both indoor and outdoor spaces during the pandemic, which prevented them from meeting friends or neighbors in the neighborhood. Social spaces continue to evolve in this regard and may come online in response to new circumstances. Online third places as virtual social spaces are similar to those proposed by Oldenburg. Since then, several studies have been conducted to determine whether online third places have similar characteristics to third places in the real world (Soukup, 2006; Parkinson et al., 2021). Online third places primarily serve a hedonistic function and promote a playful mood and sociability (Ducheneaut et al., 2007; Moore et al., 2009). Online third places can also serve as virtual communities that bring together people with similar interests (Bugliarello, 1997), share information, conduct business, communicate with each other, make friends, or play games (Rheingold, 1994; Wadhwa and Kotha, 1999).

It is widely recognized that users can satisfy their social needs by communicating and interacting with friends, family members, peers, or colleagues, or by accessing news and entertainment in online communities (Wadhwa and Kotha, 1999). Online third places are more accessible than physical (real-world) third places because they are not limited by space or time (Markiewicz, 2019). Despite the lack of a physical location, users or members in online communities can build lifelong connections with others, provided the community contains members who share their interests or lifestyles. In this way, connectedness with friends is considered valuable social capital that has been shown to be a predictor of life satisfaction (Haslam et al., 2012).

Numerous online third places have been shown to be beneficial to the well-being of older people. For example, social media chatrooms such as Instagram, Facebook, and Twitter provide easily accessible and inexpensive communication tools that engage older adults in their social networks, which can increase feelings of social connectedness and reduce loneliness (Quinn, 2019; Cotton et al., 2020). Mobile instant messaging apps such as WhatsApp or WeChat can improve older adults’ well-being by fostering better interpersonal relationships and social interactions (Bong and Chen, 2015; Lo, 2020). According to Hämmerle et al. (2020), older adults also use WhatsApp to build, strengthen, and maintain meaningful relationships. Numerous studies have also shown that older adults benefit from online learning communities. For older adults, online learning communities provide an interactive platform for lifelong learning, allowing them to learn new skills, solve problems, and engage in discussions. Lifelong learning, according to Xu et al. (2019), can improve older adults’ cognitive abilities and mental health, which in turn affects their overall well-being. Most of these studies emphasized the importance of third places in the virtual world (online third places) for older adults’ well-being, but less empirical attention has been paid to these third places in times of pandemics. Although online third places provide social opportunities for older adults, there is still much to learn about the specific types of online third places that are critical for increasing life satisfaction among older adults.

**Fig. 1:**
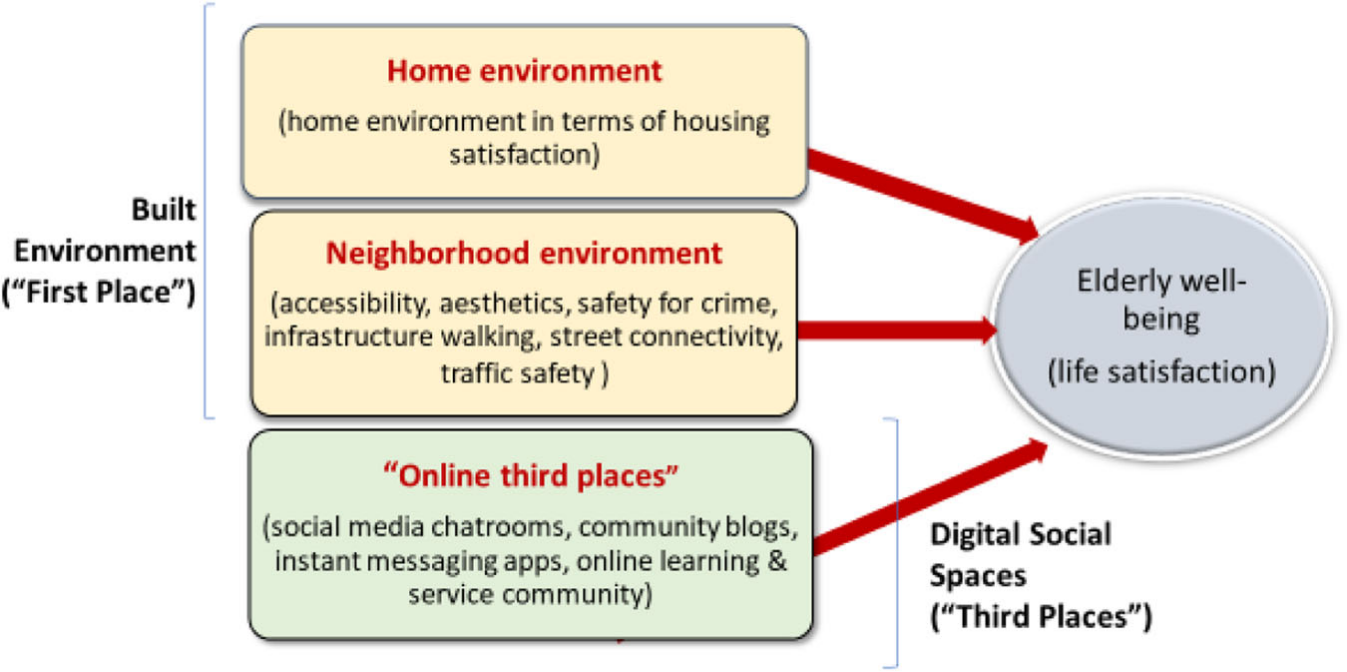
Conceptual framework of elderly well-being and environments.

With the literature supporting the relationship between the built and virtual social environments and older people’s well-being, Fig. 1 shows the home environment in terms of housing satisfaction, the neighborhood environment in terms of accessibility, esthetics, safety from crime, infrastructure walking, street connectivity, and traffic safety, along with online third places, such as social media chatrooms, online community forums or blogs, online learning communities, and messaging apps, that contribute to older adults’ well-being in Malaysia.

Given the preceding discussion, the research questions of this study were:

- To what extent does housing satisfaction influence Malaysian older adults’ life satisfaction during the epidemic?
- To what extent do neighborhood characteristics such as accessibility, esthetics, crime safety, infrastructure for walking, street connectivity, and traffic safety influence Malaysian older adults’ life satisfaction during the epidemic?
- To what extent do online third places, affect Malaysian older adults’ life satisfaction during the epidemic?

## Methods

### Area of study and respondents

For this study, a questionnaire was used in conjunction with purposive sampling to obtain the required sample size. The target population of the study is active older adults in Malaysia’s Klang Valley aged 60 years and above. The reason for selecting Klang Valley in particular is due to its dense urban environment (Tey (2012)). Klang Valley, the most developed area in Malaysia, is also known as Greater Kuala Lumpur. The Malaysian government has officially designated it as a National Key Economic Area” (NKEA). In the Klang Valley, 20% of the population is over 60 years old (CodeBlue, 2021). This number is expected to increase as Malaysia’s demographics change and the country’s population ages rapidly. For this study, the four districts of Klang Valley - Klang, Petaling, Kepong, and Wangsa Maju - were selected. In each of these four districts, 400 older adults were surveyed using the drop-off and pick-up method (DOPU) during the pandemic, which lasted from May 20, 2021, to March 20, 2022. Although 564 survey questionnaires were collected (response rate 36% of distributed questionnaires), only 500 questionnaires were included in this analysis after removing questionnaires with missing data for some questions.

In this research, the qualitative method was also used to gain insights into the experiences and opinions of the respondents and to shed light on unexpected findings from the quantitative data. Expectations related to the physical built environment and online social environment as determinants of life satisfaction during the coronavirus outbreak were the subject of in-depth interviews with seven older adults who were neighborhood leaders in the four districts of Klang Valley. In this study, leaders of residential associations and community clubs were selected as neighborhood leaders. The following are some of the interview questions: “Are you satisfied with your home?”, “What aspect of your neighborhood do you think has the most impact on your life satisfaction during the pandemic?”, “Are you familiar with social media chatrooms?”, “How often do you chat with your friends on social media during the pandemic?”, “To what extent do you think messaging apps like WhatsApp or WeChat can affect people’s happiness levels in their daily lives during the pandemic?”. The researchers took great lengths to explain the meaning of the questions sent to community leaders and the main findings of the interviews with 500 respondents. The content of the interviews was codified and divided into categories related to the built and social environment.

### Variables used in the quantitative method

The constructs of life satisfaction and neighborhood environment were rated on a four-point scale ranging from (1) strongly disagree to (4) strongly agree. Similarly, the housing satisfaction construct was measured on a Likert scale ranging from (1) very dissatisfied to (4) very satisfied. In measuring life satisfaction, five questions about older adults’ satisfaction with their life were used (Diener et al., 1985). Following Cerin et al. (2006), six aspects of neighborhood environment were measured, namely accessibility (4-item), infrastructure for walking and cycling (4-item), safety for crime (2-item), esthetics (4-item), traffic safety (4-item), and street connectivity (3-item). Five questions were asked of older adults about their level of satisfaction with their current housing conditions: the kitchen and washing areas, the size of the unit, the bathroom, the brightness inside the unit, and the type of housing unit (Savasdosara et al., 1989; Amole, 2009; Tan, 2012).

Because there was no established scale for measuring the use of online third places during the pandemic, five online third places to socialize were identified: social media chatrooms, online community forums, instant messaging apps, online learning classes, and online service communities. Respondents were asked to rate their frequency of visits for socialization on a scale of never (1) to often (4).

### Statistical analysis

In this study, the partial least squares structural equation modeling (PLS-SEM) method was used for statistical analysis. This technique is particularly useful for investigating plausible causality, developing theories, and making predictions (Hair et al., 2011). Compared to traditional covariance-based SEM, PLS is independent of small sample sizes and makes no use of distributional assumptions (Hair et al., 2011). To obtain direct, indirect, and total effects, the PLS-SEM algorithm and consistent bootstrapping procedures (5000 samples) were used.

### Reliability and validity

Several statistical procedures were used to assess the reliability and validity of the survey’s multi-item constructs. Confirmatory factor analysis was used to assess item reliability, convergent validity, internal consistency, and discriminant validity of the measurement model. According to the literature criterion, when evaluating the measurement model, the loadings should be greater than or equal to 0.5, the composite reliability (CR) should be ≥ 0.70, and the average variance extracted (AVE) should be ≥ 0.50. Table 1 shows that all AVEs were > 0.60 and all CRs were > 0.80.

**Table 1:** Measurement model.

Variable	Items	Loadings	CR	AVE
Esthetics	A1	0.804	0.936	0.785
	A2	0.923		
	A3	0.898		
	A4	0.913		
Accessibility	Acc1	0.897	0.938	0.792
	Acc2	0.923		
	Acc3	0.873		
	Acc4	0.867		
Housing satisfaction	HS1	0.833	0.926	0.716
	HS2	0.913		
	HS3	0.853		
	HS4	0.844		
	HS5	0.781		
Infrastructure for walking	ISW1	0.912	0.875	0.642
	ISW2	0.907		
	ISW3	0.663		
	ISW4	0.688		
Life satisfaction	LS1	0.896	0.949	0.788
	LS2	0.898		
	LS3	0.887		
	LS4	0.917		
	LS5	0.838		
Safety crime	S1	0.976	0.977	0.955
	S2	0.979		
Street connectivity	SC1	0.925	0.940	0.838
	SC2	0.945		
	SC3	0.875		
Traffic safety	TS2	0.860	0.911	0.774
	TS3	0.883		
	TS4	0.895		

In addition, the HTMT criteria proposed by Henseler et al. (2015) were used to assess discriminant validity. As suggested, the HTMT values for conceptually dissimilar and conceptually similar constructs should be less than 0.85 and 0.90, respectively. Table 2 shows that the HTMT values were all < 0.85. Both tables showed that respondents considered these constructs to be distinct, valid, and reliable.

**Table 2:** Discriminant validity (HTMT).

	ACC	A	S	HS	ISW	LS	SC	TS
ACC								
A	0.389							
S	0.466	0.264						
HS	0.314	0.635	0.197					
ISW	0.487	0.626	0.277	0.346				
LS	0.464	0.724	0.414	0.726	0.466			
SC	0.052	0.181	0.134	0.142	0.374	0.209		
TS	0.541	0.795	0.408	0.506	0.824	0.669	0.152	

## Results

### Structural model

Path coefficients, standard errors, *t*-values, and *p*-values for the structural model were reported as suggested by Hair et al. (2019). A combination of criteria, such as confidence intervals and effect sizes, were reported in addition to the previously mentioned criterion to test the significance of the hypotheses (see Table 3). Overall, the *R*^2^ (adjusted *R*^2^) of the model was 0.671 (0.655), which means that all predictors could explain 67.1% (65.5%) of the variance in the life satisfaction of older adults.

**Table 3:** First place, online third place and life satisfaction of the elderly.

	Std. Beta	Std. error	*t*-values	*p*-values	BCI LL	BCI UL	*f*^2^	VIF
Accessibility	0.039	0.049	0.796	0.213	−0.042	0.118	0.004	1.582
Esthetics	0.230	0.062	3.707	0.000	0.133	0.334	0.075	2.606
Safety for crime	−0.021	0.045	0.479	0.316	−0.100	0.046	0.001	1.698
Infrastructure walking	−0.165	0.067	2.472	0.007	−0.282	−0.061	0.032	3.157
Street connectivity	0.103	0.039	2.606	0.005	0.043	0.170	0.031	1.242
Traffic safety	0.155	0.074	2.096	0.018	0.044	0.285	0.026	3.377
Housing satisfaction	0.275	0.047	5.786	0.000	0.196	0.354	0.153	1.831
OP1 social media chatrooms	0.026	0.048	0.547	0.292	−0.053	0.105	0.001	1.873
OP2 community forums/blogs	0.028	0.049	0.566	0.286	−0.050	0.110	0.001	2.006
OP3 instant messaging apps	0.120	0.055	2.182	0.015	0.031	0.210	0.027	1.975
OP4 online learning/classes	0.039	0.053	0.740	0.230	−0.045	0.128	0.002	2.257
OP5 online service community	0.006	0.058	0.109	0.457	−0.094	0.096	0.000	2.186
*R*^2^	0.671							
(Adjusted *R*^2^)	0.655							

The results of Table 3 show that, all else being equal, none of the online third places was significant during the pandemic, except for instant messaging apps. The results of the internal and external environment variables of life satisfaction differed in some respects from previous studies before the epidemic, but not in others. In this study, esthetics (*b* = 0.230), walking infrastructure (*b* = −0.165), street connectivity (0.103), traffic safety (*b* = 0.155), and housing satisfaction (*b* = 0.275) were all statistically significant predictors of life satisfaction during the pandemic. However, there were no significant relationships between life satisfaction and accessibility as well as safety for the crime.

## Discussion

### Home and neighborhood environments (“First place”)

The pandemic forces people to spend more time at home. By examining how people feel about their current housing situation, it is possible to determine whether or not people’s housing needs are being addressed during the pandemic. According to this study, respondents who were generally satisfied with their housing situation had higher life satisfaction. Older adults generally prefer to live in their own homes. The structural design of the home has a direct impact on whether an older person can age in place. For some older people, their current housing situation is not always appropriate, so they are constantly reevaluating their housing conditions in light of their needs and resources. In order to meet the physical housing needs of older people, the habitability aspect of housing must be addressed by considering the changing needs and lifestyles of older people. Regardless of the epidemic, it is imperative to consider the role of housing characteristics in supporting well-being and meeting the diverse needs of older adults. As one respondent pointed out, “As we age, our housing needs become more closely tied to our health and care needs; that’s why we choose housing with senior-friendly features like wider bathrooms or non-slip bathroom floors.”

To improve the overall well-being of older adults during the coronavirus outbreak, factors affecting the neighborhood environment should be considered in addition to housing characteristics. Estimates from the model found that the esthetic appearance of the neighborhood, characterized by the presence of trees and attractive natural landmarks and buildings, was significantly related to life satisfaction. Access to natural resources such as green space close to home appears to be a factor in well-being later in life. “I prefer to live in a community with lots of fresh air and lush flora,” said one respondent. Another respondent shared this sentiment, saying, “Being in contact with a lot of greenery and a beautiful landscape gives me energy and a good feeling. That’s very important to me, especially during the pandemic when we cannot move around” There is growing recognition that well-designed natural landscapes and green spaces adjacent to and between homes are critical to making neighborhoods livable. Access to green and natural spaces can positively influence individual satisfaction (Pfeiffer and Cloutier 2016). Life satisfaction among older adults can be influenced by the presence of nature and vegetation, as shown in research from the United Kingdom and Colombia (Curl and Mason, 2019; Parra et al., 2010). Numerous studies have also demonstrated the positive effects of greenery on physical, mental, and social health (Evans, 2003; Scopelliti et al., 2016; Lachowycz and Jones, 2013).

The results in Table 3 show that, all else being equal, neighborhood permeability, as measured by street connectivity and the construction of sidewalks for pedestrians and a bicycle network, is statistically significantly associated with the well-being of the elderly. However, one relationship is positive while the other is negative. In this survey, participants generally agreed that good street connectivity in their community was associated with higher well-being. Having a high number of transit hubs and alternative routes in the neighborhood does not hurt the quality of life, according to this response. Integrated planning to promote pedestrian mobility through the construction of sidewalks and a bicycle network is an essential component of a livable and sustainable residential environment. However, the low life satisfaction reported by respondents is related to pedestrian and bicycle infrastructure. It appears that respondents do not consider their neighborhood to be a pedestrian-friendly living environment. One female respondent commented, “There are not many bikes or pedestrian-friendly paths in my neighborhood” We cannot eliminate driving because there are no well-connected, pedestrian-friendly sidewalks and no bike network in my neighborhood, said another respondent.

As driving ability declines with age, there is a need to focus more on building a pedestrian and bicycle-friendly environment, which is critical to aging in place. Well-developed sidewalks and bikeways can help reduce car dependence and promote pedestrian activity, both of which should improve the quality of life for older adults (Clarke et al., 2008, 2011). According to Curl and Mason (2019), there is a relationship between life satisfaction and the view of a dedicated pedestrian network, as older people may experience fewer falls and injuries as a result of pedestrian-friendly sidewalks (Devkota et al., 2017).

In the model of this study, traffic safety proved to be a significant predictor of life satisfaction, highlighting its importance. This significance may be related to the fact that higher levels of traffic safety increase residents’ well-being. According to a study by Balfour and Kaplan (2002), a reduction in traffic in the neighborhood could improve the quality of life of older people because it decreases the likelihood of overall functional deterioration.

The hypothesis that older adults’ well-being is related to their home’s accessibility to neighborhood amenities and services is not supported by this study. As explained by Gibler and Taltavull (2010), accessibility to neighborhood services matters to older adults, i.e., proximity to services such as public transportation, a grocery store, a restaurant, a bank, a pharmacy, medical facilities, and a post office. The fact that most people strive to avoid using local services may explain why accessibility to services shows less impact during the pandemic. There is no denying that certain habits will change as a result of the Movement Control Order (MCO) lockdown. As one respondent commented, “We stay at home because the stores are closed due to the lockdown measures.” The pandemic has made people less anxious to use public services. One respondent expressed concern about the risk of using public transportation, “I do not use public busses or trains during the pandemic because some of the passengers do not follow standard operating procedures” (SOP)”.

This study also examined how respondents perceived safety in their current neighborhood. Holding all other factors constant, neighborhood safety from crime was not significantly related to life satisfaction. It appears that older adults are concerned that safety measures in their neighborhood will not increase their life satisfaction. Some respondents made the following observation: “I do not feel safe walking on the street at night because of poor street lighting.” “Poor pedestrian lighting prevents people with low vision from walking after dark,” they added. These responses highlight the need to consider neighborhood safety issues, as older adults who consider their neighborhoods safe are more likely to participate in outdoor physical activities than older adults who consider their neighborhoods risky. In addition, Clarke et al. (2011) found that older people are more likely to engage in routine interpersonal contact when their neighborhood is safer because they are more likely to socialize with their neighbors and friends. (Yu et al., 2019). According to Curl and Mason (2019), older people are more likely to walk in the neighborhood if they feel comfortable there. Because rising crime rates can degrade the quality of the built environment and reduce community satisfaction, a crime-free neighborhood is necessary to raise people’s standard of living (Lovejoy et al., 2010).

### Online third places

As virtual social spaces facilitate the emergence and growth of online communities, virtual meeting places can become third places (Markiewicz, 2019). Online communities consist of people who may never meet in person and who communicate through computer bulletin boards and networks (Rheingold, 1994). People often spend more time in online communities for social, educational, and entertainment reasons (Rheingold, 1994; Annstrong and Hagel, 1996). It is reasonable to assume that online third places provide a virtual platform where older adults can spend their leisure time. However, the results show that most online third spaces, including social media chat rooms, online community blogs, online learning communities, and online service communities, have a negligible impact on older adults’ life satisfaction. It seems that these online third spaces have not been able to provide the Malaysian elderly with a good platform to satisfy their needs for friendship and emotional support throughout the pandemic.

The results of social media chatrooms are at odds with those of Gaia et al. (2021) and Sala et al. (2021), who found that compared to non-users in European countries, those who use more social media report being more satisfied with their lives. Similar results were found in China, where older adults who spend more time daily using social media chatrooms report feeling more of a part of their community, connected to others, and satisfied with their lives (Zhao, 2023; Zhao et al., 2021). Considering that these studies were conducted before the pandemic, this study did not find a significant relationship between Malaysian older adults’ life satisfaction and social media chatrooms during the pandemic.

The results of this study also contradict an earlier study conducted in Australia that suggested that online service communities can promote life satisfaction (Parkinson et al., 2021). Online service communities, unlike other online third places such as social media chatrooms, are typically created to support the delivery of core services. In the context of Malaysia, life satisfaction and online service communities appear to be unrelated in times of pandemic.

Blogging allows older adults to connect with people with similar interests and build an online blog community. This is especially possible with blogs that focus on health and wellness in the United States (Miller and Pole, 2010). However, according to this study, Malaysian older adults may be less accustomed to online community blogs or forums (Yu, 2021), which may make it more difficult for them to engage in online activities such as blogging.

According to a UK study, using online learning resources and participating in online learning communities can help older adults prevent cognitive decline and other age-related problems (Xiong and Zuo, 2019), which can lead to an increase in life satisfaction. However, the Malaysian study found the opposite.

As reported by Mustafa and Hamzah (2014), 66% of Malaysians use online third places, but these numbers represent Malaysians as a whole, not a breakdown. For younger Malaysians, the Internet and social media have become an integral part of life (Amurthalingam, 2023), but this is different for older adults in Malaysia. The digital divide between older and younger people still exists, even though more older people are using the Internet and social media. Compared to younger people, older adults use much fewer digital communication tools and spend less time online. According to Knowles and Hanson (2018), older people’s lack of use of digital technologies and social media is significantly influenced by several other factors, including deeply held beliefs about the benefits of technology use, broader concerns about its impact on society, and concerns about misuse of the software. There are fewer expectations for older people to “meet” in online third places. Barriers such as financial expenses, lack of confidence, and lack of expertise may prevent older people from using social media platforms for socializing (Haase et al., 2021; Knowles and Hanson, 2018). In addition, some older people turn away from virtual social venues because they value the social benefits of regular face-to-face interactions (Baker et al., 2018).

This is evident from the study data, which show that most online third places are insignificant for older people. Consistent with the findings of Vaportzis et al. (2017), older people are discouraged from using online third places because they perceive them as difficult and unsafe. Similar to Yazdani-Darki et al. (2020), older people avoid using technology to avoid cyberbullying, especially in the context of their age as ‘too old.” “I avoid going online because I don’t feel safe at all, plus I’m afraid of internet scams,” one respondent said in her responses. “I’ve no idea who is hiding behind the screen, and I could be a convenient target for their smear campaigns and lies; therefore, I still prefer to meet with my friends in person whenever possible,” she continued. Another respondent is cautious about joining online communities, believing that the connections made there are short-lived and superficial: “In my experience, relationships between members in cyberspace tend to be superficial, and lasting relationships with other people are impossible.

The only preferred online third place that Malaysian older adults would use regularly, according to this study, is an instant messaging app such as WeChat or WhatsApp. One respondent commented, “We cannot have regular meetings because of Covid-19. To make phone calls, we often use WhatsApp or WeChat. Instead of online blogs, I feel more comfortable with messaging apps.” Indeed, Malaysians frequently use the messaging apps WeChat and WhatsApp (Amurthalingam, 2023). These instant messaging apps have user-friendly, intuitive interfaces that are easy to use. This makes it easier for older adults, who may not be as tech-savvy, to use them and connect with their friends and family. In addition, these apps provide older adults with a platform to connect with their loved ones, participate in social networks, and join communities. This is especially important for older adults who are isolated or have limited mobility.

Although some older adults believe that social media platforms can help improve social support (Czaja et al., 2018) and psychological well-being (Fang et al., 2018), there are still areas that need to be filled to optimize older adults' use of online third places. In this regard, moderators, authors, or owners of chatrooms, forums, and learning and community sites should provide opportunities for interaction with users and make them feel comfortable. This can be accomplished by actively initiating threads on a variety of topics over time that allow members to share social support and improve members' life satisfaction.

## Conclusion

In recent decades, academics and planners have supported aging in place through urban planning and design. Understanding the relationship between environmental elements and the well-being of older people is critical to developing effective policies and programs to promote aging in place. However, this research was conducted before the onset of the epidemic. This study fills this gap by examining the relationship between the built environment and life satisfaction during the coronavirus pandemic.

The results of this study suggest that builders need to consider the changing needs of older people. Because they will need to spend more time at home during the pandemic, housing conditions should therefore take into account both their changing needs and how effectively the space would support them during the pandemic. People of all ages are affected by housing characteristics, but older people may be more affected than other groups. Compared to younger adults, they are more vulnerable to physical and mental health problems and more dependent on social support from the community. This study has shown that residing in a neighborhood with good-quality housing is associated with a higher likelihood of life satisfaction.

This study recognizes the importance of home environment and neighborhood characteristics during the pandemic on the life satisfaction of older adults and highlights the importance of considering these factors of aging in place when designing residential environments for older adults. To support aging in place during the pandemic, the home and neighborhood environments in which older adults live should promote independence, support social contact, and provide opportunities for safety and physical activity. It is generally believed that older adults who exhibit higher levels of life satisfaction tend to remain in their homes and communities as they age.

It is not clear whether interest-based chatrooms, local community blogs or forums, online learning courses, and online service communities can serve as online third places for older people to socialize and build social connections, which in turn could affect life satisfaction. According to this study, online third places do not foster social connections among Malaysian older adults in times of pandemic. It seems that third places developed in the virtual world cannot replace traditional communities and develop into a new type of social group. Consequently, policymakers need to find innovative ways to provide older adults with the training they need to participate in online third places, as the ability to age in place while remaining socially connected is increasingly dependent on the ability to use modern technologies.

### Recommendations and limitations

Although perceived access to amenities did not have a significant impact on life satisfaction in this study, it is necessary to consider the importance of a well-connected living environment when designing an age-friendly neighborhood. As many studies have found, easy access and proximity to amenities such as public transportation or local stores are related to older people’s well-being. It is critical to have elements of accessibility that allow older adults to stay connected to the community even after mobility limitations are lifted. Perceived neighborhood safety from crime also did not predict life satisfaction. In the spirit of promoting aging in place, it is suggested that neighborhood crime prevention efforts continue, as safe and secure neighborhoods foster a strong sense of community among residents.

Encourage older adults to seek out online third places so they can join interest groups for support, communication, and social contact, and learn new things by participating in online learning communities, which could improve their life satisfaction. Some older adults may be reluctant to seek out online third places because they lack confidence in using digital communication technologies. Therefore, it is important to help them set up their digital and online accounts and guide them through the various features of online third places. In terms of privacy and online security, they should be given instructions on how to deal with security issues related to online third places.

However, this study has some drawbacks. First, only older adults in Malaysia’s Klang Valley were used for data collection. To assess the generalizability of our findings on the influence of home, neighborhood, and online social environments on life satisfaction among older adults, more similar studies should be conducted in other parts of Malaysia in the future. Second, the focus of this study is primarily on healthy and active older adults. The needs of older adults with limited mobility should be investigated in future research.

## Data Availability

All of the data analyzed and reported in this study are publicly available and can be obtained upon request.

## References

[CR1] Aldosemani T, Radddaoui A, Shepherd C, Thompson J (2016). Second life as a third place for English language learners’ cross-cultural interaction. Q Rev Distance Educ.

[CR2] Ambrey C, Fleming C (2014). Public greenspace and life satisfaction in urban Australia. Urban Stud.

[CR3] Amole D (2009). Residential satisfaction in student housing. J Environ Psychol.

[CR4] Amurthalingam S (2023) Social media statistics for Malaysia. https://www.meltwater.com/en/blog/social-media-statistics-malaysia. Accessed 15 Feb 2023

[CR5] Anglano C, Canonico M, Guazzone M (2016). Forensic analysis of the ChatSecure instant messaging application on android smartphones. Digit Investig.

[CR6] Annstrong A, Hagel J (1996). The real value of online communities. Harv Bus Rev.

[CR7] Baker S, Warburton J, Waycott J, Batchelor F, Hoang T, Dow B, Ozanne E, Vetere F (2018). Combatting social isolation and increasing social participation of older adults through the use of technology: a systematic review of existing evidence. Australas J Ageing.

[CR8] Balfour JL, Kaplan GA (2002). Neighborhood environment and loss of physical function in older adults: evidence from the Alameda County Study. Am J Epidemiol.

[CR9] Bong WK, Chen W (2015). Mobile instant messaging for the elderly. Procedia Comput Sci.

[CR10] Bugliarello G (1997). Telecommunities: the next civilization. Futurist.

[CR11] Ceci L (2022) Mobile messaging users worldwide 2025. Statista. https://www.statista.com/statistics/483255/number-of-mobile-messaging-users-worldwide/. Accessed 18 Feb 2023

[CR12] Cerin E, Saelens BE, Sallis JF, Frank LD (2006). Neighborhood environment walkability scale: validity and development of a short form. Med Sci Sports Exerc.

[CR13] Clarke PJ, Ailshire JA, Bader M, Morenoff JD, House JS (2008). Mobility disability and the urban built environment. Am J Epidemiol.

[CR14] Clarke PJ, Ailshire JA, Nieuwenhuijsen EE, de Kleijn-de Vrankrijker MW (2011). Participation among adults with disability: the role of urban environment. Soc Sci Med.

[CR15] CodeBlue (2021) Two-thirds of Klang Valley elderly vaccinated against Covid-19. https://codeblue.galencentre.org/2021/06/24/khairy-two-thirds-of-klang-valley-elderly-vaccinated-against-covid-19/ Accessed 10 Nov 2022

[CR16] Cotton SR, Schuster AM, Seifert A (2020). Social media use and well-being among older adults. Curr Opin Psychol.

[CR17] Curl A, Mason P (2019). Neighborhood perception and older adults’ wellbeing: does walking explain the relationship in deprived urban communities?. Transp Res Part A.

[CR18] Czaja SJ, Boot WR, Charness N, Rogers WA, Sharit J (2018). Improving social support for older adults through technology: findings from the PRISM randomized controlled trial. Gerontologist.

[CR19] Devkota S, Anderson B, Soiza RL, Myint PK (2017). Prevalence and determinants of frailty and associated comorbidities among older Gurkha welfare pensioners in Nepal. Geriatr Gerontol Int.

[CR20] Diener E, Emmons RA, Larsen RJ, Griffin S (1985). The satisfaction with life scale. J Pers Assess.

[CR21] Diener E, Suh EM, Lucas RE, Smith HL (1999). Subjective well-being: three decades of progress. Psychol Bull.

[CR22] Djebuarni R, Al-Abed A (2000). Satisfaction level with neighborhood in low income public housing in Yamen. Prop Manag.

[CR23] Ducheneaut N, Moore RJ, Nickell E (2007). “Virtual ‘third places’: a case study of sociability in massively multiplayer games. Comput Support Coop Work.

[CR24] Evans GW (2003). The built environment and mental health. J Urban Health.

[CR25] Fan V, Cheong R (2021) MCO, CMCO, RMCO, CMCO again: regulations and sops—coronavirus (COVID-19)—Malaysia. Welcome to Mondaq. https://www.mondaq.com/operational-impacts-and-strategy/1022936/mco-cmco-rmco-cmco-again-regulations-and-sops Accessed 10 Nov 2022

[CR26] Fang Y, Chau AK, Wong A, Fung HH, Woo J (2018). Information and communicative technology use enhances psychological well-being of older adults: the roles of age, social connectedness, and frailty status. Aging Ment Health.

[CR27] Feng J, Tang S, Chuai X (2018). The impact of neighborhood environment on quality of life of elderly people: evidence from Nanjing, China. Urban Stud.

[CR28] Fleming CM, Manning M, Ambrey CL (2016). Crime, greenspace and life satisfaction: an evaluation of the New Zealand experience. Landsc Urban Plan.

[CR29] Gaggero A, Fernández-Pérez A, Jiménez-Rubio D (2022). Effect of the COVID-19 pandemic on depression in older adults: a panel data analysis. Health Policy.

[CR30] Gaia A, Sala E, Cerati G (2021). Social networking sites use and life satisfaction. A quantitative study on older people living in Europe. Eur Soc.

[CR31] Gibler KM, Taltavull P (2010). Using preferences for international retiree housing market segmentation. J Prop Res.

[CR32] Grini ISB, Ueland Ø (2023). How families’ use of digital technology can be a tool for reducing loneliness and improving food intake among older adults. J Ageing Longev.

[CR33] Haase KR, Cosco T, Kervin L, Riadi I, O’Connell ME (2021). Older adults’ experiences with using technology for socialization during the COVID-19 pandemic: cross-sectional survey study. JMIR Aging.

[CR34] Hair JF, Ringle CM, Sarstedt M (2011). PLS-SEM: indeed a silver bullet. J Mark Theory Pract.

[CR35] Hair JF, Risher J, Sarstedt M, Ringle CM (2019). When to use and how to report the results of PLS-SEM. Eur Bus Rev.

[CR36] Hämmerle V, Pauli C, Braunwalder R, Misoch S (2020) WhatsApp’s influence on social relationships of older adults. In: Proceedings of the 6th international conference on information and communication technologies for ageing well and e-Health. SCITEPRESS, pp. 93–98

[CR37] Haslam SA, Reicher SD, Levine M (2012) When other people are heaven, when other people are hell. In: Jetten J, Haslam C, Haslam S (eds) The social cure: identity, health, and wellbeing. Psychology Press, pp.157–174

[CR38] Henseler J, Ringle C, Sarstedt M (2015). A new criterion for assessing discriminant validity in variance-based structural equation modeling. J Acad Mark Sci.

[CR39] James P, Hart JE, Banay RF, Laden F, Signorello LB (2017). Built environment and depression in low-income African Americans and Whites. Am J Prev Med.

[CR40] Knowles B, Hanson VL (2018). Older adults’ deployment of ‘distrust’. ACM Trans Comput–Hum Interact.

[CR41] Kunduraci AC (2017). Lighting design for the aging eyes. Int J Sci Technol.

[CR42] Lachowycz K, Jones AP (2013). Towards a better understanding of the relationship between greenspace and health: development of a theoretical framework. Landsc Urban Plan.

[CR43] Langlais M, Vaux DE (2022). Establishing and testing a quantitative measure for evolving third-place characteristics. Int J Technol Hum Interact.

[CR45] Leigh-Hunt N, Bagguley D, Bash K, Turner V, Turnbull S, Valtorta N, Caan W (2017). An overview of systematic reviews on the public health consequences of social isolation and loneliness. Public Health.

[CR46] Lo OW (2020). Exploring the use of mobile instant messaging among middle-aged adults in social relationships maintenance with family and friends. Asian J Media Commun.

[CR47] Lovejoy K, Handy S, Mokhtarian P (2010). Neighborhood satisfaction in suburban versus traditional environment: an evaluation of contributing characteristics in eight California neighborhoods. Landsc Urban Plan.

[CR48] Maas J, Verheij RA, Groenewegen PP, de Vries S, Spreeuwenberg P (2006). Green space, urbanity, and health: how strong is the relation?. J Epidemiol Community Health.

[CR49] Markiewicz E (2019). Third places in the era of virtual communities. Stud Perieget.

[CR50] Marzo RR, Vinay V, Bahari R, Chauhan S, Ming DAF, Nelson Fernandez SFA, Johnson CCP, Thivakaran AQA, Rahman MM, Goel S (2021). Depression and anxiety in Malaysian population during third wave of the COVID-19 pandemic. Clin Epidemiol Glob Health.

[CR51] McArthur JA, White AF (2016). Twitter chats as third places: conceptualizing a digital gathering site. Soc Media Soc.

[CR52] Miller EA, Pole A (2010). Diagnosis blog: checking up on health blogs in the blogosphere. Am J Public Health.

[CR53] Moore RJ, Gathman ECH, Ducheneaut N (2009). From 3D space to third place: the social life of small virtual spaces. Hum Org.

[CR54] Mou JB (2020) Study on social media marketing campaign strategy—TikTok and Instagram Thesis, Massachusetts Institute of Technology. https://dspace.mit.edu/handle/1721.1/127010 Accessed 17 Feb 2023

[CR55] Mustafa SE, Hamzah A (2014) Online social networking as a third place: usage in Malaysia. In: Kertas kerja biennial convention of the Pacific and Asian communication. Universitas Padjadjaran, Indonesia

[CR56] Oldenburg R (1999). The great good place: cafes, coffee shops, bookstores, bars, hair salons and other hangouts at the heart of a community.

[CR57] Park S, Lee S (2016). Age-friendly environments and life satisfaction among South Korean elders: person-environment fit perspective. Aging Mental Health.

[CR58] Parkinson J, Schuster L, Mulcahy R (2021). Online third places: supporting well-being through identifying and managing unintended consequences.. J Serv Res.

[CR59] Parra DC, Gomez LF, Sarmiento OL, Buchner D, Brownson R, Schimd T, Lobelo F (2010). Perceived and objective neighborhood environment attributes and health-related quality of life among the elderly in Bogota, Colombia. Soc Sci Med.

[CR60] Pfeiffer D, Cloutier S (2016). Planning for happy neighborhoods. J Am Plann Assoc.

[CR61] Quinn K (2019). Social media and social well-being in later life. Innov Aging.

[CR62] Rheingold H (1994) The virtual community: homesteading on the electronic frontier. Addison-Wesley, Reading

[CR63] Robinson S, Deshano C (2011). Citizen journalists and their third places: what makes people exchange information online (or not)?. J Stud.

[CR64] Ryff CD (2014). Psychological well-being revisited: advances in the science and practice of eudaimonia. Psychother Psychosom.

[CR65] Sala E, Cerati G, Gaia A (2021). Are social media users more satisfied with their life than non-users? A study on older Italians.. Ageing Soc.

[CR66] Sallis JF, Cerin E, Conway TL, Adams MA, Frank LD, Pratt M, Salvo D, Schipperijn J, Smith G, Cain KL, Davey R, Kerr J, Lai PC, Mitáš J, Reis R, Sarmiento OL, Schofield G, Troelsen J, Van Dyck D, De Bourdeaudhuij I, Owen N (2016). Physical activity in relation to urban environments in 14 cities worldwide: a cross-sectional study. Lancet.

[CR67] Savasdosara T, Tips WEJ, Suwannodom S (1989). Residential satisfaction in private estates in Bangkok, a comparison of low-cost housing estates and determinant factors. Habitat Int.

[CR68] Scopelliti M, Carrus G, Adinolfi C, Suarez G, Colangelo G, Lafortezza R, Panno A, Sanesi G (2016). Staying in touch with nature and well-being in different income groups: the experience of urban parks in Bogotá. Landsc Urban Plan.

[CR70] Sirgy MJ, Cornwell T (2002). How neighborhood features affect quality of life. Soc Indic Res.

[CR92] Sixsmith A, Horst BR, Simeonov D, Mihailidis A (2022). Older people’s use of digital technology during the COVID-19 pandemic. Bull Sci Technol Soc.

[CR71] Soukup C (2006). Computer-mediated communication as a virtual third place: building Oldenburg’s great good places on the world wide web. New Media Soc.

[CR72] Tan TH (2012). Housing satisfaction in medium- and high-cost housing: the case of Greater Kuala Lumpur, Malaysia. Habitat Int.

[CR73] Tan TH (2016). Neighborhood satisfaction: responses from residents of green township in Malaysia. Int J Hous Mark.

[CR74] Tan TH, Lee JH (2022) Residential environment, third place and wellbeing in Malaysian older adults. Soc Indic Res 10.1007/s11205-021-02856-8

[CR75] Tan TH, Lee WC (2023) Life satisfaction and perceived and objective neighborhood environments in a green-accredited township: quantile regression approach. Cities 134 10.1016/j.cities.2023.104196

[CR76] Tey NP (2012). Internal migration in the Klang Valley of Malaysia: issues and implications. Malays J Chin Stud.

[CR77] Tsuchiya-Ito R, Slaug B, Ishibashi T (2019). The physical housing environment and subjective well-being among older people using long-term care services in Japan. J Hous Elder.

[CR78] Tu JC, Lin KC, Chen HY (2020). Investigating the relationship between the third place and the level of happiness for seniors in Taiwan. Int J Environ Res Public Health.

[CR79] Vaportzis E, Clausen MG, Gow AJ (2017). Older adults perceptions of technology and barriers to interacting with tablet computers: a focus group study. Front Psychol.

[CR80] Wadhwa A, Kotha S (1999) A note on virtual communities. https://sureshkotha.files.wordpress.com/2018/05/virtual_communities1.pdf. Accessed 10 Nov 2022

[CR81] Xia N, Li H (2018). Loneliness, social isolation, and cardiovascular health. Antioxid Redox Signal.

[CR82] Xiong J, Zuo M (2019). Older adults’ learning motivations in massive open online courses. Educ Gerontol.

[CR83] Xu H, Yang R, Qi X (2019). Association of lifespan cognitive reserve indicator with dementia risk in the presence of brain pathologies. JAMA Neurol.

[CR84] Yazdani-Darki M, Rahemi Z, Adib-Hajbaghery M, Izadi FS (2020). Older adults’ barriers to use technology in daily life: a qualitative study. Nurs Midwifery Stud.

[CR85] Yu CY (2021) Make technology senior-friendly. New Straits Times (December 11). https://www.nst.com.my/opinion/columnists/2021/12/753284/make-technology-senior-friendly. Accessed 15 Feb 2023

[CR86] Yu R, Cheung O, Lau K, Woo J (2017). Association between perceived neighborhood walkability and walking time, wellbeing and loneliness in community-dwelling older Chinese people in Hong Kong. Int J Environ Res Public Health.

[CR87] Yu S, Liu Y, Cui C, Xia B (2019). Influence of outdoor living environment on elders’ quality of life in old residential communities. Sustainability.

[CR88] Zaccaria D, Guaita A, Vaccaro R, Cassanova G, Abbondanza S, Pettinato L, Cerati G, Rolandi E, Sala E (2020). Assessing the impact of social networking site use on older people’s loneliness and social isolation. A randomized controlled trial: the aging in a networked society-social experiment study (ANS-SE). Contemp Clin Trials Commun.

[CR89] Zhang Z, Zhang J (2017). Perceived residential environment of neighborhood and subjective well-being among the elderly in China: a mediating role of sense of community. J Environ Psychol.

[CR90] Zhao L (2023). The effects of mobile social media use on older migrants’ social integration and life satisfaction: use types and self-esteem perspective. Soc Sci Comput Rev.

[CR91] Zhao L, Liang C, Gu D (2021). Mobile social media use and trailing parents’ life satisfaction: social capital and social integration perspective. Int J Aging Hum Dev.

