# Correction: Assessing the significance of first place and online third places in supporting Malaysian seniors’ well-being during the pandemic

**DOI:** 10.1057/s41599-023-01753-4

**Published:** 2023-05-19

**Authors:** Teck Hong Tan, Izian Idris

**Affiliations:** 1 Xiamen University Malaysia, School of Economics and Management, Bandar Sansuria, Malaysia; 2 Sunway University, Sunway Business School, Bandar Sunway, Malaysia

**Keywords:** Environmental studies, Economics, Health humanities

Correction to: *Humanities and Social Sciences Communications* 10.1057/s41599-023-01655-5, published online 07 April 2023.

A correction has been made in this article’s abstract to amend a word ‘parties’ to ‘places’. This error that could have resulted in confusion on the part of the reader.

The original abstract text read:

The enforced lockdowns and social distancing measures associated with COVID-19 may have influenced older adults’ preferences towards their homes and neighborhoods as well as social spaces. One objective of this research is to determine whether home and neighborhood environments (“first place”) affect how satisfied older adults are with their lives during the epidemic. This study also examined the extent to which social spaces that exist in the virtual world (“online third places”) affect older adults’ life satisfaction when they would have to practice risk-averse behaviors in times of pandemic. To collect data, this study analyzed the responses of 500 active older adults and conducted in-depth interviews with seven older adults who served as neighborhood leaders in Klang Valley, Malaysia. The study found that there is a direct relationship between older adults’ satisfaction with their current housing and their overall life satisfaction during the pandemic. Similarly, having a quality neighborhood nearby increases the likelihood of living a satisfied life during the pandemic. Most online third parties, with the exception of instant messaging apps, do not appear to provide older adults with an adequate platform to interact with their friends, participate in social networking, and join communities for emotional support during the pandemic. The findings and recommendations of this study would be very useful in developing effective interventions to promote aging in place during the coronavirus outbreak.

The revised text reads:

The enforced lockdowns and social distancing measures associated with COVID-19 may have influenced older adults’ preferences towards their homes and neighborhoods as well as social spaces. One objective of this research is to determine whether home and neighborhood environments (“first place”) affect how satisfied older adults are with their lives during the epidemic. This study also examined the extent to which social spaces that exist in the virtual world (“online third places”) affect older adults’ life satisfaction when they would have to practice risk-averse behaviors in times of pandemic. To collect data, this study analyzed the responses of 500 active older adults and conducted in-depth interviews with seven older adults who served as neighborhood leaders in Klang Valley, Malaysia. The study found that there is a direct relationship between older adults’ satisfaction with their current housing and their overall life satisfaction during the pandemic. Similarly, having a quality neighborhood nearby increases the likelihood of living a satisfied life during the pandemic. Most online third places, with the exception of instant messaging apps, do not appear to provide older adults with an adequate platform to interact with their friends, participate in social networking, and join communities for emotional support during the pandemic. The findings and recommendations of this study would be very useful in developing effective interventions to promote aging in place during the coronavirus outbreak.

